# Endoscopic MIS-TLIF with Destandau’s system: leveraging endoscopy with conventional instruments

**DOI:** 10.3171/2024.1.FOCVID23216

**Published:** 2024-04-01

**Authors:** Ashutosh Kumar, Arun Kumar Srivastava, Jayesh Sardhara, Anant Mehrotra, Kamlesh Bhaisora, Raj Kumar

**Affiliations:** Department of Neurosurgery, Sanjay Gandhi Post Graduate Institute of Medical Sciences, Lucknow, Uttar Pradesh, India

**Keywords:** endoscopic, lumbar interbody fusion, minimally invasive spine surgery, spondylolisthesis

## Abstract

This presentation showcases an endoscopic minimally invasive spine surgery (MISS) technique for lumbar interbody fusion. Significantly expanding the scope of Destandau’s system within MISS, it serves as a pivotal link to unilateral biportal endoscopy (UBE) for endofusion. The method involves minimally invasive transforaminal lumbar interbody fusion (MIS-TLIF) using a 4-mm rigid endoscope through Destandau’s system. With the widespread familiarity with Destandau’s system and the absence of specialized instrument requirements, this approach is easily adoptable, particularly in resource-limited centers. The favorable clinical and radiological outcomes underscore the effectiveness of this technique, propelling the role of endoscopy in MISS, particularly in endofusion.

The video can be found here: https://stream.cadmore.media/r10.3171/2024.1.FOCVID23216

## Transcript

This video illustrates a minimally invasive spine surgery technique for addressing L5–S1 listhesis with paracentral disc protrusion. This technique offers the advantage of unilateral biportal endoscopy while mitigating the need for a wet medium.^1–3^ Moreover, it does not necessitate additional instruments beyond those used in the standard microscopic minimally invasive transforaminal lumbar interbody fusion.

### 0:45 Clinical Presentation.

The 33-year-old lady presented with severe low backache with left L5 radiculopathy following a recent bike accident. Examination revealed weakness in the left extensor hallucis longus (EHL) and sensory loss along the left L5 dermatome.

### 0:59 Radiological Imaging.

Standing x-ray lateral view indicated grade 1 L5–S1 anterolisthesis with a pars interarticularis fracture. Based on spinopelvic parameters, the patient was classified as type 2 according to the modified Spine Deformity Study Group (mSDSG) classification.^4^ On MRI, there was a left L5–S1 paracentral disc with foraminal extension and a cranially migrated portion.

### 1:21 Diagnosis and Surgical Plan.

The patient was diagnosed with L5–S1 grade 1 listhesis with L5–S1 migrated paracentral disc with foraminal extension. She was planned for MIS-TLIF using a 4-mm rigid endoscope, followed by percutaneous pedicle screw fixation (PPSF).

### 1:36 Patient Positioning.

The patient was positioned prone, under general anesthesia.

### 1:41 Marking for Anatomical Landmarks.

Skin markings were made under fluoroscopic guidance. The midline, a line along the lateral edge of the pedicles, and the upper endplates were marked. A 16-gauge needle was inserted as a guide for docking the Destandau’s system, pointing to the L5–S1 disc space on the contralateral side.

### 1:58 Incision.

A 2-cm longitudinal incision was marked along the pedicle line, starting from the S1 pedicle and extending toward the L5 pedicle for Destandau’s system insertion.

### 2:09 Docking of Destandau’s System.

Docking was done and the level was confirmed with C-arm. Initial docking is done at the spinolaminal junction.

### 2:16 Exposure of the Laminofacet Junction.

Lateral exposure to the laminofacet junction is performed by removing the soft tissue and a small amount of muscle with monopolar and bipolar cautery.

### 2:24 Facet Joint Exposure.

The facet joint is thus exposed. It is called the "valley between two mountains" view, showing the L5 inferior facet, S1 superior facet, and the intervening joint space.

### 2:34 Laminectomy.

The inferior edge of the L5 lamina is removed with No. 3 Kerrison punch.

### 2:39 Three-Point Drilling.

The rest of the lower lamina is drilled at three strategic points using a 3-mm diamond burr. Firstly at the lower margin. Secondly at the junction of the pars interarticularis and the inferior facet by working laterally from the first point. This would help resection of the L5 inferior facet. And lastly at the root of the spinous process to allow over-the-top decompression of the spinal canal and the contralateral nerve root. The remaining bone is removed with a Kerrison punch.

### 3:08 Inferior Facet Resection

As the pars interarticularis and the inferior facet junction was removed with the Kerrison’s punch, the whole of the inferior facet could be disconnected. The disconnection of the L5 inferior facet entails a careful separation from its ligamentous attachment. Utilizing biopsy forceps, it was divided into two halves so as to deliver it through the outer sheath. These bone pieces can be used to pack the polyetheretherketone (PEEK) cage to improve fusion rates.

### 3:34 Removal of the S1 Superior Facet.

Now the facetal surface of the S1 facet is seen. This facet is removed using No. 3 Kerrison punch, working medial to lateral until we reach the S1 pedicle. The surface is smoothened using a burr while avoiding any injury to the pedicle.

### 3:49 Resection of Ligamentum Flavum.

The ligamentum flavum is then removed in piecemeal. It will expose the underlying epidural fat. It should be retained till all the bony work is completed to avoid a dural tear.

### 4:00 Dural Exposure.

Removal of the epidural fat exposes the thecal sac.

### 4:07 Exposure of the Traversing Nerve Root.

Working laterally to the thecal sac in a craniocaudal direction, the fat and soft tissue are dissected to expose the traversing nerve root.

### 4:25 Exposure of the Disc.

The traversing nerve root is gently retracted medially at its shoulder to expose the disc space. The Destandau’s retractor is used to keep the nerve root retracted. The engorged epidural veins are coagulated and cut.

### 4:40 Confirmation of Disc Level.

Using a marking needle, the disc level is confirmed under the C-arm.

### 4:45 Discectomy.

The lower part of the Kambin’s triangle was thus exposed. Using a 15 No. blade, the annulus is cut. The nucleus pulposus and part of the annulus fibrosus are removed using biopsy forceps. Care is taken to remove all the herniated disc, including the migrated portion.

### 5:07 Insertion of the Second Port.

A 1.5-cm horizontal incision was made at the marked level for inserting the L5 percutaneous pedicle screw (PPS). This incision is deepened till the fascia. The first of the dilators for PPS is inserted, and sounding is done with the Destandau’s outer sheath to confirm alignment. The dilator is further navigated into the disc space under endoscopic vision. Triangulation of the instruments is thus achieved.

Successively, the second dilator is then inserted, followed by the third dilator sheath, which is used for screw insertion. It is inserted until its lower margin is just visible by the endoscope and left in situ to act as a new port. The endoscope is then inserted in this new port. The inner sheath of the Destandau’s system is removed, allowing the outer sheath to serve as an access port for the larger instruments to be introduced into the disc space.

### 6:07 Endplate Preparation.

Larger shavers can now be inserted through the Destandau’s outer sheath to prepare the disc space like in tubular discectomy. The endoscope can be freely advanced into the disc space to inspect the status of the endplate preparation.

### 6:26 Sizer Followed by Cage Insertion.

The sizer for the cage was inserted under endoscopic vision, thus preventing nerve injury. Due to the transforaminal corridor, only a slight retraction of the traversing nerve root is required when using a large cage.

A 14 × 28–mm PEEK cage was inserted and its position was confirmed on the C-arm. For adequate visualization of the nerve root on the medial aspect of the cage, a 30° endoscope is required at times. The dislodged bone chips from the cage and remaining disc fragments were removed. At the end of the procedure, the L5–S1 left lateral recess and foramina were completely free.

### 7:10 Over-the-Top Decompression.

The spinal canal and the contralateral nerve root were decompressed by the over-the-top technique. The thecal sac and the traversing nerve root (S1) can be seen to be well pulsatile and supple, confirming no compression. Afterward, L5–S1 PPSF was done.

### 7:29 Postoperative Status.

The patient was ambulated 12 hours after the surgery. She was now able to walk without pain. There was a complete improvement in the left EHL power by the 3rd week of follow-up. Postoperative CT affirmed correction of the listhesis with optimal implant position. The total operative time was 4.5 hours, with an estimated blood loss of 300 ml. The patient was discharged in 5 days with significant improvement in the VAS scores.

The technique has its own benefits, nuances, and limitations. It expands the spectrum of Destandau’s system and is a bridge to unilateral biportal endoscopy, thus increasing the role of endoscopy in minimally invasive spine surgeries.

## Disclosures

The authors report no conflict of interest concerning the materials or methods used in this study or the findings specified in this publication.

## Author Contributions

Primary surgeon: A Kumar. Editing and drafting the video and abstract: A Kumar. Critically revising the work: all authors. Reviewed submitted version of the work: A Kumar, Srivastava, Bhaisora, R Kumar. Approved the final version of the work on behalf of all authors: A Kumar. Supervision: Srivastava, Mehrotra, R Kumar.

## Supplemental Information

### Patient Informed Consent

The necessary patient informed consent was obtained in this study.
